# Subthalamic nucleus deep brain stimulation driven by primary motor cortex γ2 activity in parkinsonian monkeys

**DOI:** 10.1038/s41598-022-10130-1

**Published:** 2022-04-20

**Authors:** Olivier Darbin, Nobuhiko Hatanaka, Sayuki Takara, Nobuya Kaneko, Satomi Chiken, Dean Naritoku, Anthony Martino, Atsushi Nambu

**Affiliations:** 1 Division of System Neurophysiology, National Institute for Physiological Sciences, Okazaki, Aichi Japan; 2 Department of Neurology, University South Alabama College of Medicine, 307 University Blvd, Mobile, AL 36688 USA; 3 Department of Physiological Sciences, SOKENDAI (Graduate University for Advanced Studies), Okazaki, Aichi Japan; 4 Department of Physiology, Faculty of Medecine, Kindai University, Osaka-Sayama, Osaka, Japan; 5 Department of Neurosurgery, University South Alabama College of Medicine, Mobile, AL USA

**Keywords:** Parkinson's disease, Parkinson's disease

## Abstract

In parkinsonism, subthalamic nucleus (STN) electrical deep brain stimulation (DBS) improves symptoms, but may be associated with side effects. Adaptive DBS (aDBS), which enables modulation of stimulation, may limit side effects, but limited information is available about clinical effectiveness and efficaciousness. We developed a brain-machine interface for aDBS, which enables modulation of stimulation parameters of STN-DBS in response to γ2 band activity (80-200 Hz) of local field potentials (LFPs) recorded from the primary motor cortex (M1), and tested its effectiveness in parkinsonian monkeys. We trained two monkeys to perform an upper limb reaching task and rendered them parkinsonian with 1-methyl-4-phenyl-1,2,3,6-tetrahydropyridine. Bipolar intracortical recording electrodes were implanted in the M1, and a recording chamber was attached to access the STN. In aDBS, the M1 LFPs were recorded, filtered into the γ2 band, and discretized into logic pulses by a window discriminator, and the pulses were used to modulate the interval and amplitude of DBS pulses. In constant DBS (cDBS), constant stimulus intervals and amplitudes were used. Reaction and movement times during the task were measured and compared between aDBS and cDBS. The M1-γ2 activities were increased before and during movements in parkinsonian monkeys and these activities modulated the aDBS pulse interval, amplitude, and dispersion. With aDBS and cDBS, reaction and movement times were significantly decreased in comparison to DBS-OFF. The electric charge delivered was lower with aDBS than cDBS. M1-γ2 aDBS in parkinsonian monkeys resulted in clinical benefits that did not exceed those from cDBS. However, M1-γ2 aDBS achieved this magnitude of benefit for only two thirds of the charge delivered by cDBS. In conclusion, M1-γ2 aDBS is an effective therapeutic approach which requires a lower electrical charge delivery than cDBS for comparable clinical benefits.

## Introduction

Deep brain stimulation (DBS) targeting the subthalamic nucleus (STN) successfully treats motor symptoms related to Parkinson’s disease (PD) and dopaminergic replacement therapy, i.e., bradykinesia, tremor, and dyskinesia^1^. Patients are conventionally treated with constant DBS (cDBS), which delivers continuous stimulation of constant parameters, such as stimulus frequency, amplitude, and pulse duration. To improve efficacy and efficiency and decrease side effects of DBS, adaptive DBS (aDBS) has been alternatively proposed. The concept of aDBS is to optimize the delivery of electrical pulses as a function of biomarkers presupposed to reflect the patient’s needs in therapeutics^2–5^.

At the clinical level, one of the potential biomarkers is β band (13–30 Hz) activity in the basal ganglia, especially the STN. The β band activity allegedly opposes the realization of movement as it exhibits higher power during the hypokinetic state and lower power during active movement in healthy^6,7^ and PD^8–10^ subjects. Oscillatory activity in the cortico-basal ganglia-thalamocortical circuitry is generally envisaged by a push–pull model between the slow β band and the fast γ band activity^11^; although both β^12,13^ and γ^14^ activities have been reported to be up-regulated in PD. Therefore, aDBS driven by β activity in the STN (STN-β aDBS) would deliver more stimulation in the hypokinetic state and therefore could treat hypokinesia more effectively. However, it would fail to deliver therapeutic benefits when the patients attempts movements, which is arguably when the patient needs it the most.

Therefore, an alternative approach would be to design aDBS system aimed to increase the benefits of electrical stimulation when patients engage in voluntary movement^15^. One of the electrophysiological biomarker related to voluntary movements could be γ band (> 30 Hz) activity, especially γ2 band (80–200 Hz) activity in the primary motor cortex (M1). The γ band activity reflects processes considered to enhance the realization of movement. Activity in γ band increases while preparing and executing voluntary movement in normal^16^ and PD conditions^17,18^. M1-γ activity correlates positively with tremor^19^ in PD patients and with dyskinetic symptoms of L-dopa induced dyskinesia^20^. The clinical observations raised above support the view that M1-γ activity^21–27^ might be an alternative biomarker for aDBS (M1-γ2 aDBS).

**Figure 1:**
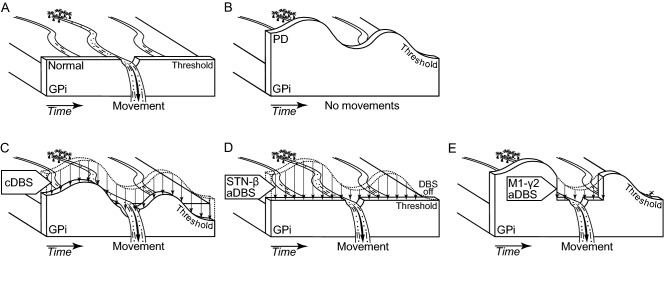
The “dam model” of basal ganglia functions^28^ and the expected effects of STN-DBS using constant and adaptive paradigms. (**A**) Normal state: In the healthy state, only the selected motor plans pass over the threshold of the internal pallidal (GPi) function, causing the intended movement, while other plans are blocked. (**B**) Parkinson’s disease (PD) condition: In parkinsonism, the threshold of the GPi function is elevated, which causes the blockade of all motor plans, resulting in hypokinesia. (**C**) cDBS in PD: Constant deep brain stimulation (cDBS) delivers constant stimulation to the subthalamic nucleus (STN), continuously lowers the threshold of the GPi function, and allows the motor plans to cause the intended movements. (**D**) STN-β aDBS in PD: Adaptive DBS (aDBS) is modulated by β activity in the STN, which is a biomarker of hypokinesia. STN-β aDBS delivers stimulation when hypokinetic state occurs, temporary lowers the threshold of the GPi function, and increases the likelihood for selection of motor plans during this time window. (**E**) M1-γ2 aDBS in PD: aDBS is modulated by γ2 activity in the primary motor cortex (M1), which is a biomarker of movement-related activity. M1-γ2 aDBS delivers stimulation when motor plans are presupposed being generated in the M1, lowers the threshold of the GPi function, and increases the likelihood for selection of motor plans in this time window.

At the neurophysiological level, the pathophysiological mechanism of PD and beneficial mechanism of STN-DBS are still under debate. One over the several favored explanatory frameworks is the “dam model” of the basal ganglia functions (Fig. 1)^28^. Under this framework, the basal ganglia process as activation function for different decisions reflecting information related to motor and cognitive plans. According to this view, the basal ganglia implement plans only when a criterion level of activation is reached. One anatomo-functional object relevant to carry this criterion level of activation is the internal pallidal (GPi) function. Again speculative^29^, the GPi function dynamically modulates the competition between the cortically generated plans via a threshold based system (Fig. 1A). In PD condition, the threshold of the GPi function is higher, which decreases the likelihood for implementation of the cortically generated plans (Fig. 1B)^30^. During STN-DBS in PD condition, the threshold is decreased, which increases the likelihood for a plan to be selected (Fig. 1C,D,E)^31,32^. In this view, cDBS may decrease by an offset the threshold of the GPi function (Fig. 1C), and STN-β aDBS may decrease the threshold of the GPi function depending of the hypokinetic state fluctuations in time (Fig. 1D). However, it can be argued that nominal competition between plans presupposes a temporal organization between the generation of the plans in the cortex and the modulation of the threshold of the GPi function (Fig. 1A). For this reason and in PD condition, M1-γ aDBS may lower the threshold of the GPi function when plans are presupposed to be generated in the M1 (Fig. 1E) and, therefore, improves the activation function of the basal ganglia.

In the present study, we developed a brain-machine interface to positively modulate the rate and amplitude of the stimulation train of STN-DBS as function of γ2 activity captured in the local field potentials (LFPs) of the M1. The M1-γ2 aDBS was tested in two PD monkeys during a simple motor task. This study was aimed to establish a proof of feasibility and clinical benefits. A comparison to cDBS is provided to highlight the dynamical aspect of M1-γ2 aDBS, although this pre-clinical study was not designed for a robust comparison of efficacy between aDBS and cDBS. Our collective data show that M1-γ2 aDBS is clinically beneficial in PD condition.

## Materials and methods

### Animals

The experimental protocol was approved by the Institutional Animal Care and Use Committees of the National Institutes of Natural Sciences (Okazaki, Japan) and in accordance with ARRIVE guidelines. All methods were carried out in accordance with relevant guidelines and regulations. We used 2 female Japanese monkeys (*Macaca fuscata*; monkey A, 5.0 kg; monkey B, 5.4 kg). Body weight and daily living activities were monitored throughout the study.

### Behavioral task and training

**Figure 2:**
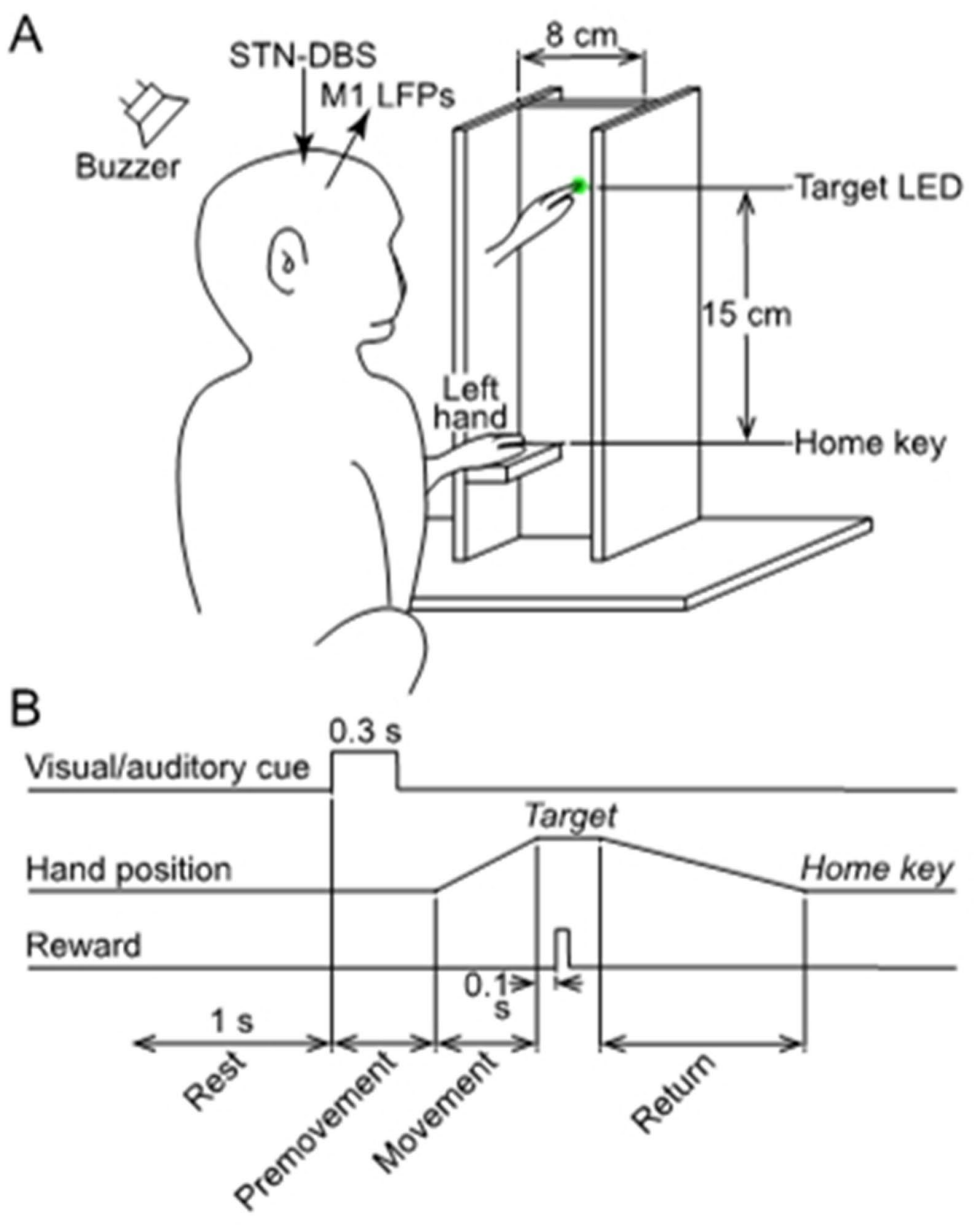
Motor task in parkinsonian monkeys. (**A**) Vertical reaching movement task using an upper limb. The monkey rested her hand on a horizontal platform (Home key) with the elbow at 90 degrees of flexion. A green target light emitting diode (LED) was embedded in a vertical platform 15 cm above the home key. In response to the simultaneous lighting of the LED and sounding of the buzzer, the monkey was required to reach the LED with its upper limb. When the monkey touched the LED, it was rewarded with a sip of water. After the reward was received, the monkey returned her hand to the home key for the next trial. The home key and target LED were equipped with infrared optoelectrical sensors to monitor hand movements. (**B**) The timing of the motor task and the definition of Rest, Premovement, Movement, and Return periods.

A simple vertical hand reaching task was used. The monkeys sat facing a plastic tower secured in front of the monkey’s chair (Fig. 2A). A green light emitting diode (LED) was embedded as a visual target in the tower at eye level. A horizontal platform was secured as a home key at the base of the tower, 15 cm below the LED, and enabled the monkey to rest her hand comfortably with the elbow flexed at 90 degrees. Home key and target LED were equipped with infrared optoelectrical sensors (Keyence, Osaka, Japan) to monitor hand movement.

The task was started after the subject rested her hand on the home key for at least 1 s (Fig. 2B). When cued by the visual and auditory signals (0.3 s), the monkey was required to reach and touch the target with her hand within 6 s. Successful completion of this task was rewarded by a sip of water 0.1 s after touching the target. The monkey was allowed 12 s to return her hand to the home key. When the monkey returned her hand to the area near the home key, the experimenter positioned her hand on the home key to initiate the next trial. We defined 4 periods in the task: (1) Rest was 1 s period before the visual and auditory stimuli; (2) Premovement began when the visual and auditory stimuli were triggered and ended when her hand moved off the home key; (3) Movement began when the monkey moved her hand off the home key and ended when she touched the target; and (4) Return began when the monkey removed her hand from the target and ended when she returned her hand to or near the home key (Fig. 2B).

Monkeys were trained 5 days a week for 4 months prior MPTP treatment. We defined a series as 25 trials with successful reach to target, with or without returning to home key. Subjects were deprived of water for 24 h before sessions, and water intake was adjusted with fresh fruits and subcutaneous fluid as needed.

### Surgery for head fixation and electrode implantation

Under general anesthesia with ketamine hydrochloride (5–8 mg/kg body weight, i.m.), xylazine hydrochloride (0.5–1 mg/kg, i.m.), and propofol (6–7 µg/ml, blood concentration, i.v.), surgery was performed to affix pipes to the skull for securing the head to a stereotaxic frame during experiments^33,34^. After 10 days, the cortical recording electrodes were implanted. After securing the head in the stereotaxic frame under general anesthesia with ketamine hydrochloride (5–8 mg/kg body weight, intramuscular) and xylazine hydrochloride (0.5–1 mg/kg, intramuscular), the skull was removed over the M1 contralateral to the hand used for task performance. The forelimb regions of M1 were identified with electrophysiological mapping^33,34^. The cortical recording electrodes were made of 200 µm-diameter Teflon-coated stainless-steel wire (California Fine Wire Co, USA). The wires were paired with matching impedance (≤ 1 k Ohm) for optimal common mode rejection^35^. They were implanted with an inter-electrode distance of 2 mm into layers III-V of the M1 forelimb region for later LFP recording and stabilized mechanically with acrylic resin. A rectangular plastic chamber that covered M1 was fixed to the skull with acrylic resin.

### Induction of parkinsonism

The monkeys were treated with 1-methyl-4-phenyl-1,2,3,6-tetrahydropyridine (MPTP; Sigma, St Louis, MO, USA) to induce moderate to severe parkinsonism with asymmetric severity. After induction of general anesthesia with ketamine hydrochloride (5–8 mg/kg body weight, i.m.), xylazine hydrochloride (0.5–1 mg/kg, i.m.), and propofol (6–7 µg/ml, blood concentration, i.v.), the external, internal, and common carotid arteries, contralateral to the hand used for task performance, were dissected at the neck region. The external carotid artery was clamped temporarily, and MPTP dissolved in saline (2 mg/mL) was injected into the common carotid artery (monkey A, 0.6 mg/kg; monkey B, 1.3 mg/kg). Later, they received additional intramuscular MPTP injections (monkey A, 0.6 mg/kg/injection, 5 injections) or additional intravenous MPTP injections (monkey B, 0.8 and 0.7 mg/kg, 2 injections) thorough the saphenous vein under general anesthesia with ketamine hydrochloride (5–8 mg/kg body weight, i.m.) and xylazine hydrochloride (0.5–1 mg/kg, i.m.). The total dose of MPTP received by monkey A was 3.6 mg/kg and monkey B was 2.8 mg/kg. Electrophysiological recordings in parkinsonian state were started at 5–8 weeks after the last treatment, when parkinsonian symptoms had stabilized for 2 weeks. The severity of parkinsonism was scored on the side contralateral to the carotid artery MPTP injection using the primate parkinsonian rating scale (maximum score indicating worst symptoms, 20 points)^36^. Monkeys responded to treatment with L-dopa and carbidopa (200–500 mg/day, p.o.).

### Cortical electrophysiological recording and conversion to stimulation pulses

**Figure 3:**
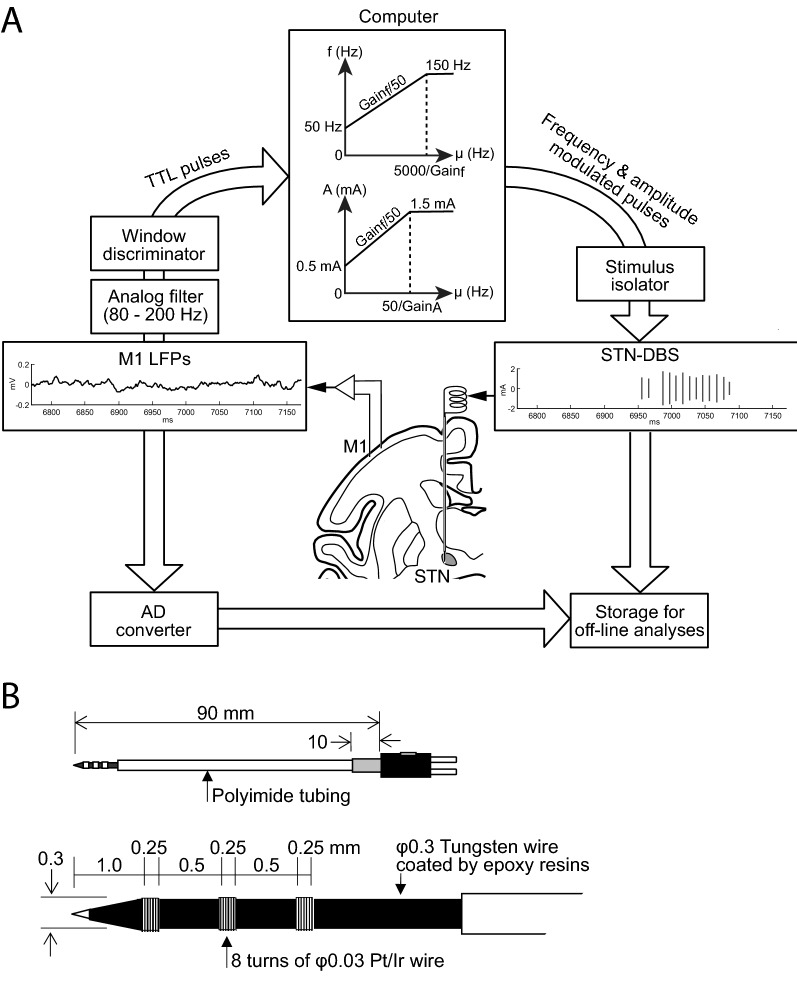
Experimental setup for aDBS driven by M1-local field potentials (LFPs). (**A**) Procedure of aDBS. LFPs were recorded in the M1, amplified, filtered (γ2 band, 80–200 Hz), and converted to transistor-transistor logic (TTL) pulses using a window discriminator that detected LFPs with amplitude > 75th percentile and duration > 0.5 ms. The frequency of the TTL pulse was used to control the amplitude and frequency of the electrical stimulation delivered to the DBS electrode in the STN via an analog stimulus isolator. (**B**) DBS electrode in the STN. Top, entire electrode. Bottom, electrode tip. Pt/Ir, alloy of platinum and iridium.

Behavioral task and electrophysiological recordings were performed in drug-off state (at least 12 hours after withdrawal).The monkey was seated in the monkey chair with the head restrained in a soundproof room shielded with copper nets to block external electromagnetic noise. The head amplifier (Cerebus, Blackrock Microsystems, Salt Lake City, UT) was placed 10 cm from the recording chamber and powered by direct current (12 V) amplified LFPs from cortical recording electrodes in the M1. From there, the signal was directed toward two distinct circuits (Fig. 3A). The first circuit consisted of the brain-machine interface aimed to modulate STN-DBS parameters as a function of γ2 activity from the M1 through the online processing system. The second circuit consisted of the data recording setup aimed to digitize and store data from the head stage, the brain-machine interface, the stimulator, and behavioral device for the off-line analyses of the experimental results^26^ through the offline processing system.

For the brain-machine interface, the M1-LFPs were amplified (x 10K, 50-300 Hz, MEG-6116/AB610J, Nihon Kohden, Tokyo, Japan) and filtered (FV-664, NF Corporation, Yokohama, Japan) to extract γ2 activity (80-200 Hz) (Fig. 3A). After preparing the primate for a recording session and prior to starting the first trial series, the M1-LFPs were recorded for 15 min while the monkey was free to move her arms, legs and trunk. This allowed the experimenter to determine the maximum oscillation amplitude in the γ2 band. A window discriminator (EN-611 J, Nihon Kohden) detected γ2 oscillations with amplitude > the 75th percentile of the maximum γ2 oscillation amplitude and duration > 0.5 ms.

The transistor-transistor logic (TTL) pulses generated by the window discriminator as a function of the γ2 band activity were routed to a computer through a digital input and analog output card (PCI6713, National Instruments, Austin, TX) for further processing. The instantaneous frequency of the γ2-driven pulses was averaged over a 50 ms window to modulate the frequency (*f*) and amplitude (*A*) of the stimulation train according to (Fig. 3A):

$$f = f_{0} \left( { f_{0} < 150} \right)$$

$$f = 150\left( {150 \le f_{0} } \right)$$

$$fo = Threshold_{f} + \frac{{\mu \left( {TTL\;pulse} \right)}}{{Threshold_{f} }}*Gain_{f}$$

and

$$A = A_{0} \left( {A_{0} < 1.5} \right)$$

$$A = 1.5\left( {1.5 \le A_{0} } \right)$$

$$Ao = Threshold_{A} + \frac{{\mu \left( {TTL\;pulse} \right)}}{{Threshold_{f} }}*Gain_{A}$$

where *μ(TTL pulse)* was the average rate of TTL pulses on a 50 ms window, *Threshold*_*f*_ was the threshold for frequency (set at 50 Hz), *Threshold*_*A*_ was the threshold for amplitude (set at 0.50 mA), *Gain*_*f*_ was the gain of frequency, and *Gain*_*A*_ was the gain of amplitude. The *Gain*_*f*_ and *Gain*_*A*_ were determined to maintain frequency and amplitude less than the defined high limits. Pulses with frequency *f*, amplitude *A*, and pulse width 60 µs were generated by the computer through a digital input and analog output card (National Instrument, PCI6713) and delivered to the DBS lead through an analog isolator (BSI-950, Dagan, Minneapolis, MN). The pulse width was set for both aDBS and cDBS at 60 µs because it did not result in artifact on M1-LFPs and is used in clinical application. We set the frequency range between 50 and 150 Hz because there is controversy about the clinical benefit associated with low-frequency stimulation^37–40^. Preliminary experiments showed that constant stimulation with amplitude 1 mA resulted in optimal performance. When stimulation exceeded 2 mA, performance decreased, and above 3 mA muscle pulling and dyskinesia were observable. These side effects were interpreted as electrical current spread toward adjacent areas of the STN such as the internal capsule. Therefore, the stimulation amplitude was set at 1 mA for cDBS and ranged from 0.5 to 1.5 mA for aDBS. We compared results with aDBS, cDBS (frequency, 100 Hz; amplitude, 1 mA; pulse width, 60 µs), and DBS-OFF (without DBS).

### Stimulation of the STN

The DBS electrodes were tungsten wire electrodes coated by epoxy resins (Fig. 3B; OM210-033a, Unique Medical, Tokyo, Japan) with a distal tip composed of a high impedance electrode (~ 500 kΩ) suitable for multiunit recordings. Additionally, these electrodes included 3 proximal contacts (inter-tips distance, 0.75 mm) with low impedance (~ 10 kΩ) suitable for stimulation. The DBS electrode was inserted either vertically or obliquely (tilted anteriorly by 36 degrees from the vertical in the sagittal plane) to the STN along the same trajectory in each experimental session. The dorso-lateral motor subregion of the STN was identified by sensory responses to passive joint movements of the arm using multiunit recording from the high impedance tip contact. The two proximal contacts were placed in the dorso-lateral motor subregion of the STN and used for bipolar stimulation.

### Postprocessing of LFPs recorded from the M1

For the off-line analyses, the M1-LFPs were recorded as bipolar signals (anterior minus posterior LFP), filtered between 3 and 200 Hz using a double reverse Butterworth filter (− 3 dB frequency of 200 Hz with 0 phase shift), stored on the computer, and down-sampled to 500 Hz. Signals were inspected visually and those with more than 3% artifacts in duration were discarded. The M1-LFPs were analyzed first with DBS-OFF condition to define the dynamics of the β, γ1, and γ2 frequency bands. We postulated that change in oscillatory activity related to the task should be band specific, of small amplitude and with high dynamics. We used a dynamic autoregressive model based on a Kalman smoother for tracking the instantaneous frequency in the β (13–30 Hz), γ1 (30–80 Hz) and γ2 (80–200 Hz) frequency bands of the M1 LFPs^26,41,42^.

**Figure 4:**
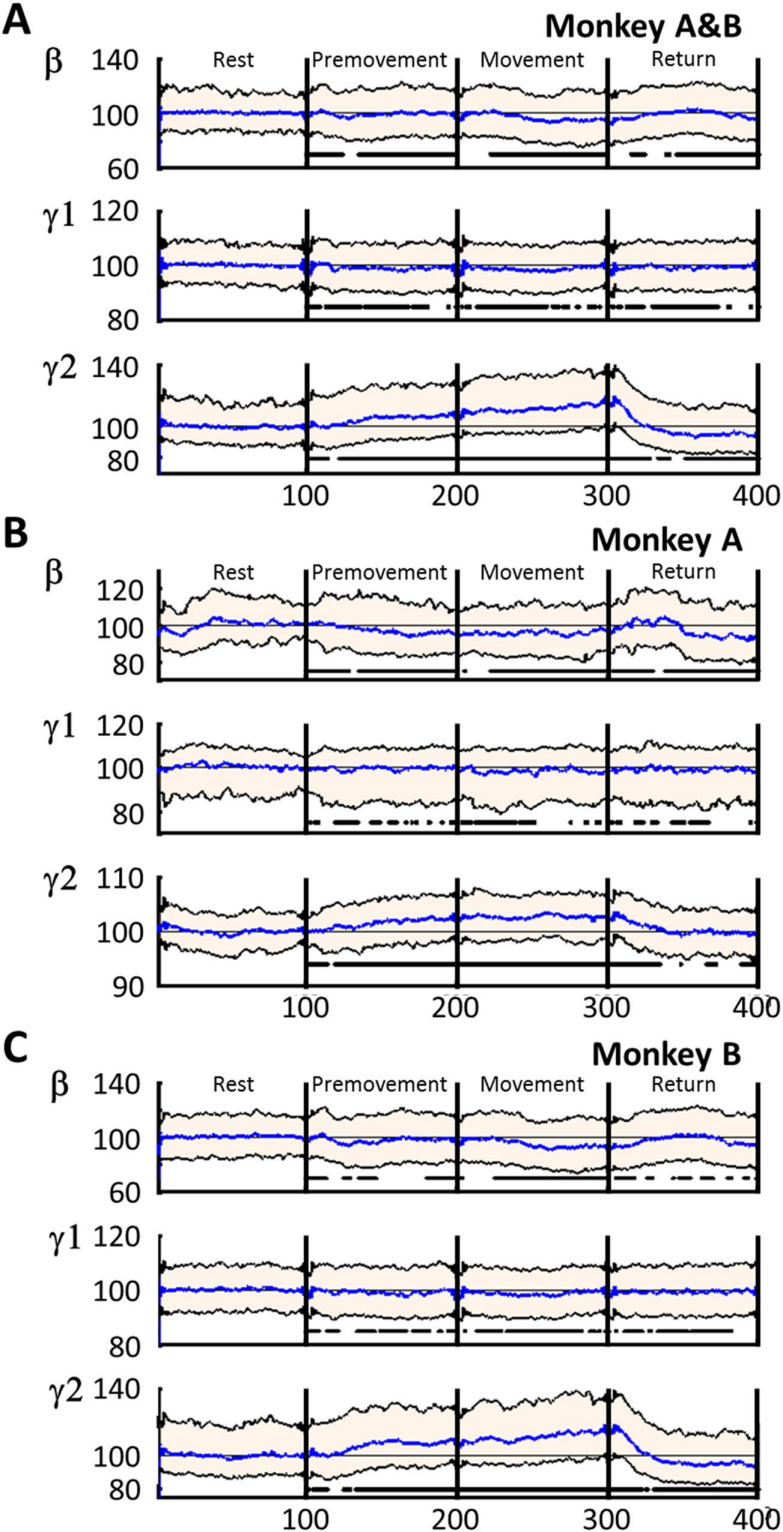
The β (13–30 Hz), γ1 (30–80 Hz), and γ2 (80–200 Hz) band activity changes during motor task periods (Rest, Premovement, Movement, and Return) with DBS-OFF. (**A**) Monkeys A and B combined. (**B**) Monkey A. (**C**) Monkey B. The activity in each band was calculated using the Kalman filter and normalized to the median activity during Rest (% of base line, BL). Time was normalized to 100 time units in each period (total, 400 relative time units). Purple, median; blue, median activity during the Rest period; black borders of pink area, interquartile range (25th to 75th percentiles); horizontal, interrupted thick black lines in the bottom indicate significant differences from the Rest period (*P* < 0.05).

The instantaneous frequencies were investigated during the 4 task periods which were aligned and normalized in time (100 points per period). The concatenation of the 100 points/period enabled synchronized trials in time for each period (Fig. 4). Graphical representations of the instantaneous frequencies and statistical analyses were performed on the synchronized trials. In PD state, the M1-LFPs were analyzed by their power in the β, γ1, and γ2 frequency bands^26^ for comparison to the method based on Kalman filter. Power spectrum density was calculated over the time defined by each task period for each trial. The power spectrum for each period was normalized to the Rest period.

### Pulse train analyses

For each period, the STN-DBS pulse trains were characterized by the duration between pulses and their amplitudes. The central tendency and dispersion of these features were used to determine changes in the stimulation train between periods and across DBS paradigms. The central tendency was estimated by the median, and the dispersion was estimated by the median absolute deviation. The electrical charge Q, expressed in Coulomb (C) per second, delivered by the isolator was calculated according to:

$${\text{Q}} = {\text{intensity}} \times {\text{pulse}}\;{\text{width}} \times {\text{frequency}}$$

### Statistical analyses

All data were reported as median and percentile range (25th-75th percentiles). Comparisons between data from different conditions were performed using the nonparametric Kruskal–Wallis and Mann–Whitney tests for pairwise comparisons with *P* < 0.05^43^ adjusted by the Benjamini–Hochberg correction^44^. Page trend test^45^ was applied to the time series of instantaneous frequencies to test the null hypothesis that the ordered series was monotonic (aka. no trend) vs. the alternative hypothesis that the series increased with *P* = 0.95.

## Results

### Severity of parkinsonian symptoms and task performance

After MPTP treatment, the two monkeys had cardinal signs for parkinsonism including akinesia, bradykinesia, rigidity, and flexed posture, more severe on the side contralateral to the carotid artery MPTP injection. The primate parkinsonian rating scale scores were stable throughout the experimental period (Table 1). Both parkinsonian monkeys responded to oral administration of L-dopa and carbidopa (L-dopa, 200 mg/d) with decreases in parkinsonian scores (monkey A, 4; monkey B; 6), but hyperkinetic symptoms were observed at higher doses (L-dopa > 500 mg/d). The two subjects were able to perform 1–6 series of 25 trials per session. Our data set includes 2583 trials (monkey A, 1112; monkey B, 1471) performed in DBS-OFF. The rate for successfully reaching the target was 87.5% (monkey A, 92.2%; monkey B, 86.6%; Table 2), and for successfully returning to the home key was 61.0% (monkey A, 90.0%; monkey B, 55.7%). The duration of each period is as follows (Table 1): Premovement, 546 ms (range, 420–833 ms); Movement, 408 ms (292–632 ms); and Return, 998 ms (627–1001 ms).

**Table 1:** Primate parkinsonian rating scale score and durations of the task periods of parkinsonian monkeys in DBS-OFF.

	Primate Parkinsonian Rating Scale score^a^	Duration median and range (25th-75th) in ms		
		Premovement	Movement	Return
Monkey A & B		546 (420–833)	408 (292–632)	998 (627–1001)
Monkey A	9/20 (Tremor, 0; Posture, 1; Gait, 2; Bradykinesia, 2; Balance, 1; Gross motor^b^, 2; Defense reaction, 1)	524 (462–600)	576 (451–789)	627 (614–637)
Monkey B	11/20 (Tremor, 0; Posture, 2; Gait, 2; Bradykinesia, 2; Balance, 1; Gross motor^b^, 2; Defense reaction, 2)	601 (323–1437)	296 (247–368)	1001 (999–1571)

The durations of Premovement, Movement, and Return periods were expressed as median and range (25th-75th percentile) in ms.

^a^Rated on the side contralateral to the carotid artery injection of MPTP. Maximum score (20) indicating worst possible symptoms.

^b^Gross motor skill in the upper limb.

**Table 2:** Success rates for the task with DBS-OFF, adaptive DBS (aDBS), and constant DBS (cDBS).

		DBS-OFF (%)	aDBS (%)	cDBS (%)
Monkeys A & B	Reach	87.5	91.5	91.5
	Return	61.0	75.9	80.7
Monkey A	Reach	92.2	99.1	96.1
	Return	90.0	98.7	96.1
Monkey B	Reach	86.6	83.2	86.3
	Return	55.7	50.9	63.0

Ratio (%) of successful trials with reaching the target (Reach) or returning to the home key (Return) among trials attempted.

### M1 LFPs during task in DBS-OFF

First, we investigated which band activity can be used as a biomarker for movements among the β, γ1, and γ2 frequency bands in the M1. Instantaneous frequency analysis of M1-LFPs in DBS-OFF showed that β, γ1, and γ2 powers were altered along the task periods (Fig. 4). There was a trend for β activity to decrease during the Premovement and Movement periods (Page’s test, *P* > 0.99) but not during the Return period (Page’s test, *P* < 0.01) (Fig. 4A). The γ1 did not show a robust monotonic trend during the Premovement period (Page’s test*, P* < 0.01) but there was a downward trend during the Movement period (Page’s test, *P* > 0.99) and no trend during the Return period (Page’s test, *P* < 0.55) (Fig. 4B). The γ2 exhibited a trend for increase during Premovement and Movement periods (Page’s test *P* > 0.99) but a trend for decrease during the Return period (Page’s test *P* > 0.99) (Fig. 4C).

**Figure 5:**
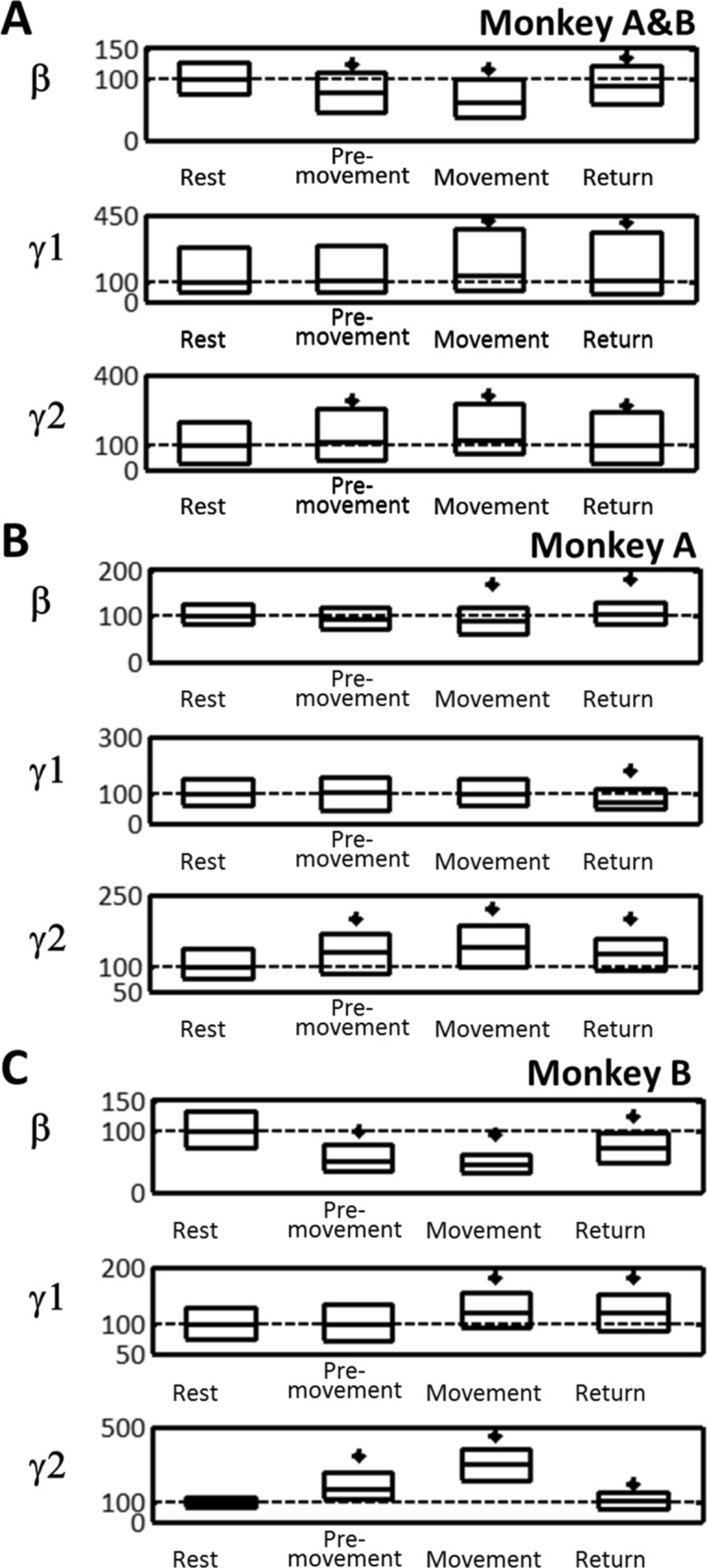
Power spectrum density in β, γ1, and γ2 bands during motor task periods (Rest, Premovement, Movement, and Return) with DBS-OFF. (**A**) Monkeys A and B combined. (**B**) Monkey A. (**C**) Monkey B. Data are expressed as percents of the median during the Rest period. Box plots indicate median and interquartile range (25th-75th percentiles). * *P* < 0.05, significantly different from Rest.

Power spectrum density during each task period (Fig. 5A) showed that median β power was reduced during the Premovement, Movement, and Return periods (*P* < 0.001). The median γ1 power for both monkeys combined was unchanged during the Premovement period but increased during the Movement and Return periods, however the median γ1 power in monkey B was unchanged during the Movement period (*P* > 0.5). The median γ2 power was increased during the Premovement, Movement, and Return periods in each individual monkey (*P* < 0.0002) (Fig. 5B&C). These changes in the dynamics and median power of γ2 suggest that M1-γ2 activity is a suitable biomarker for movements in parkinsonian condition. With the exception of γ1 activity, Kalman filter and FFT analyses were in agreement.

### Pulse intervals and amplitudes of aDBS

**Figure 6:**
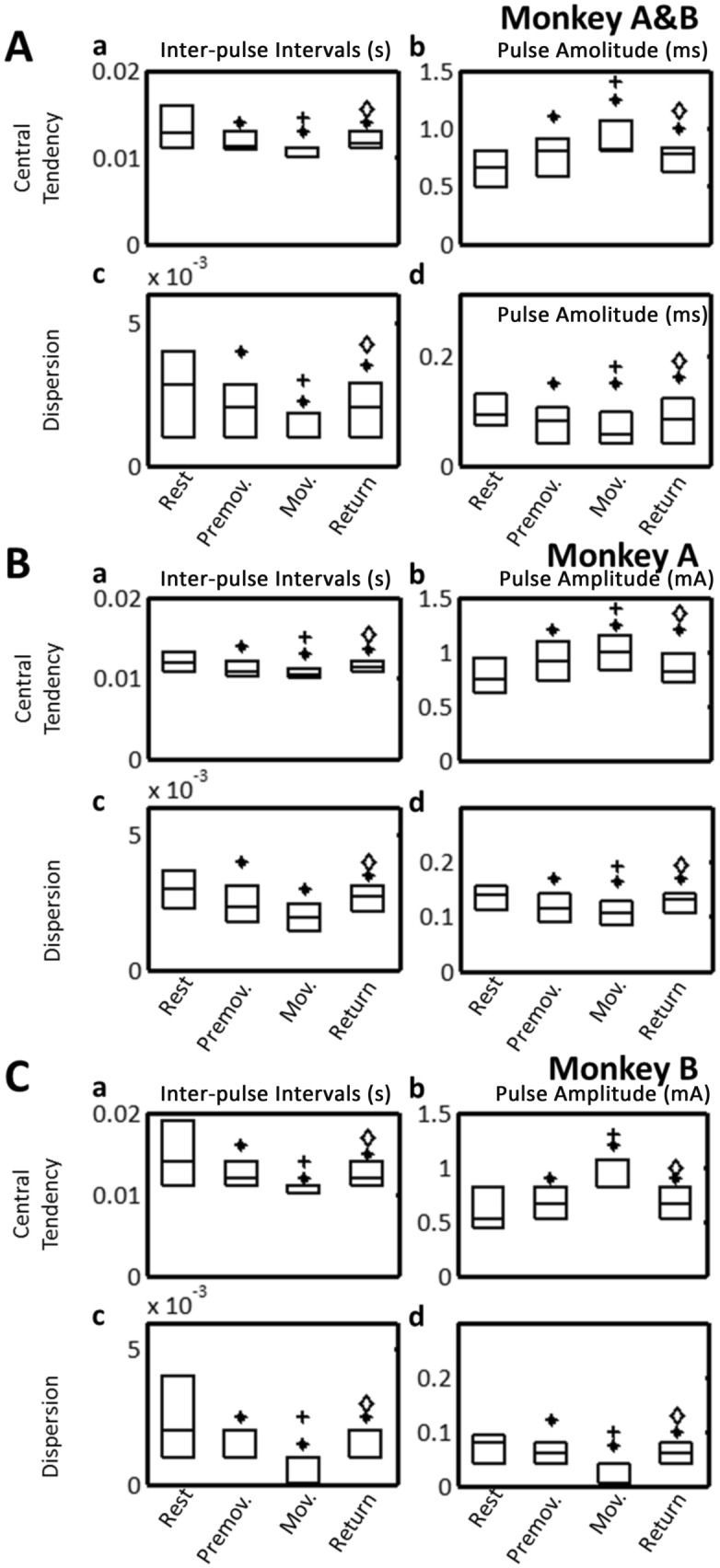
aDBS: central tendency (upper) and dispersion (lower) of stimulus intervals (left) and amplitudes (right) of train stimulation pulses during motor task periods (Rest, Premovement, Movement, and Return). (**A**) Monkeys A and B combined. (**B**) Monkey A. (**C**) Monkey B. Central tendency was estimated by the median; dispersion was estimated by the median absolute deviation. Box plots indicate median and interquartile range (25th-75th percentiles). *P < 0.05, significantly different from Rest; ^+^*P* < 0.05, significantly different from Premovement; ^◊^*P* < 0.05, significantly different from Movement.

aDBS modulated the pulse intervals and amplitudes during the task (both features, *P* < 0.001) (Fig. 6A). During the Rest period, the pulse interval was 0.012 s (range, 0.011–0.016 s), and the amplitude was 0.653 mA (0.488–0.801 mA). Pulse intervals decreased during the Premovement and Movement periods (-12.05% and -13.61%, *P* < 0.001, Fig. 6Aa), and amplitudes increased during these periods (+ 23.55% and 25.09% *P* < 0.001, Fig. 6Ab). In comparison to the Movement period, pulse intervals increased during the Return period (+ 4.35%, *P* < 0.001) but remained below values observed during the Rest period (-9.68%, *P* < 0.001, Fig. 6Aa). Pulse amplitudes decreased during the Return period (- 4.80%, *P* < 0.001) but remained above that during the Rest period (+ 19.35%, *P* < 0.001, Fig. 6Ab).

The dispersions for the pulse intervals (Fig. 6Ac) and amplitudes (Fig. 6Ad) were modulated during the task. The dispersion of the pulse interval decreased during the Premovement (− 29.41%, *P* < 0.001) and Movement (− 64.71%, *P* < 0.001) periods, and increased during the Return period (50.01%, *P*  < 0.001) remaining above that during the Rest period (29.41%, *P* < 0.001, Fig. 6Ac). The dispersion of the stimulus amplitude decreased during the Premovement (by 13%, *P* < 0.001) and Movement (by 38%, *P* < 0.001) periods. It increased during the Return period (by 33%, *P* < 0.001) and remained 10% below its value at the Rest period (*P* < 0.001, Fig. 6Ad). Changes in the central tendency and dispersion of stimulus intervals and amplitudes were similar between the two monkeys (Figs. 6B&C).

**Figure 7:**
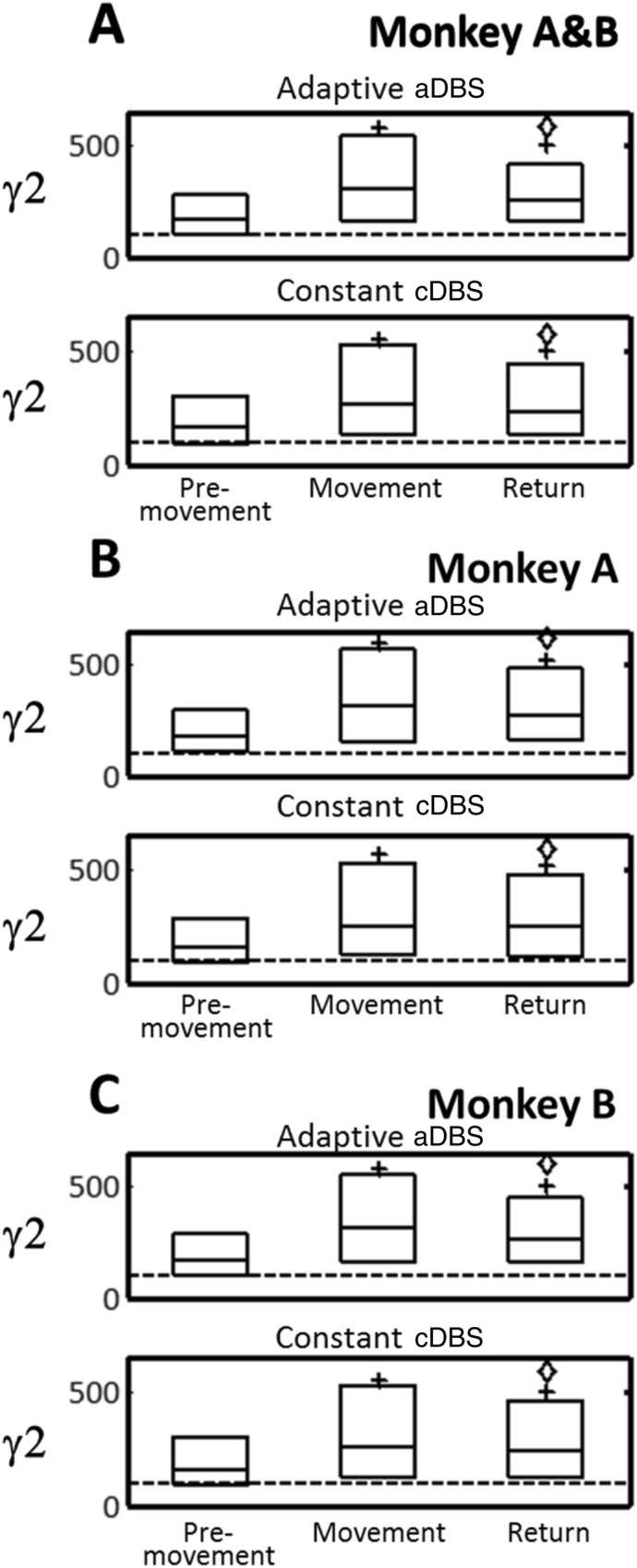
Power spectrum density in γ2 band during motor task periods (Premovement, Movement, and Return) with aDBS (upper) and cDBS (lower). (**A**) Monkeys A and B combined. (**B**) Monkey A. (**C**) Monkey B. Data are expressed as percents of the median during Rest. Box plots indicate median and interquartile range (25th-75th percentiles). ^+^*P* < 0.05, significantly different from Premovement; ^◊^*P* < 0.05, significantly different from Movement.

The M1 γ2 activity was increased during the Movement period and decreased during the Return period with aDBS and cDBS (*P* < 0.001) (Fig. 7A) in both monkey A (Fig. 7B) and monkey B (Fig. 7C).

### Behavioral changes by a DBS and cDBS

Success rates to reach the target were similar in both aDBS and cDBS (91.5%, Table 2) and success rate to return to the home key was higher with cDBS (75.9% vs. 80.7%). Monkey B was mostly responsible for this finding. The duration of each task period was compared between aDBS and cDBS (Table 3). With aDBS, the Premovement and Movement durations were reduced (− 20.1% and 15.8% *P* < 0.001), but the Return duration was unchanged (*P* > 0.05). With cDBS, the Premovement and Movement durations were reduced (−  12.0% and − 10.0%, *P* < 0.001), and the Return duration was increased (+ 6.5%, *P* < 0.001). aDBS and cDBS were clinically beneficial during the Premovement and Movement periods. aDBS, in comparison to cDBS, was found to be slightly more efficient during the Premovement (8.0% difference, *P* < 0.001) and Movement (5.8% difference, *P* < 0.01) periods. Differences in clinical effects between aDBS and cDBS appear during the Return period, with an increase duration in monkey B (by 33.2%, *P* < 0.001) but not in monkey A (*P* > 0.05).

**Table 3:** Duration of the Premovement, Movement, and Return periods with DBS-OFF, aDBS, and cDBS.

	DBS-OFF % (25th-75th)	aDBS % (25th-75th)	*P*_1_	*P*_2_	cDBS % (25th-75th)	*P*_1_
**Monkeys A & B**						
Premovement	100.0 (71.5–189.5)	79.9 (59.7–128.3)	< 0.0001	< 0.0001	88.0 (65.8–128.7)	< 0.0001
Movement	100.0 (75.9–168.4)	84.2 (66.7–129.1)	< 0.0001	< 0.0001	90.0 (72.8–136.8)	< 0.0001
Return	100.0 (99.7–102.3)	100.0 (70.4–113.3)	> 0.05	< 0.0001	106.5 (99.8–124.4)	< 0.0001
**Monkey A**						
Premovement	100.0 (85.8–132.4)	85.3 (75.2–97.6)	< 0.0001	< 0.0001	90.7 (77.2–109.8)	< 0.0001
Movement	100.0 (76.6–121.5)	82.6 (63.9–111.8)	< 0.0001	< 0.0001	89.7 (71.7–113.9)	< 0.0001
Return	100.0 (99.8–100.2)	102.0 (99.8–103.6)	< 0.001	> 0.05	100.2 (99.8–109.8)	> 0.05
**Monkey B**						
Premovement	100.0 (54.7–214.3)	73.8 (57.7–184.8)	< 0.0001	> 0.05	79.9 (54.6–189.5)	< 0.02
Movement	100.0 (73.9–333.3)	84.7 (68.4–315.4)	< 0.001	< 0.0001	90.0 (73.8–327.5)	< 0.001
Return	100.0 (44.7–178.4)	88.4 (54.7 -136.1)	< 0.005	< 0.0001	133.2 (100.1–191.4)	< 0.0001

Durations of the Premovement, Movement, and Return periods were expressed as percentage ratios and ranges (25th-75th percentile) to values in DBS-OFF. *P*_1_, *P* values in post hoc comparisons to DBS-OFF; *P*_2_, *P* values in post hoc comparisons to cDBS.

During cDBS, the electrical charge delivered by the isolator was 6 µC per second. aDBS delivered only 53.7% (26.5–73.5%, *P* < 0.001) during the Rest period and 75.7% (69.7–106.6%, *P* < 0.05) during the Movement period (Table 4) of the constant charge delivered by cDBS.

**Table 4:** Electrical charge delivered in aDBS during the Rest, Premovement, Movement, and Return periods relative to that in cDBS.

	Rest % (25th-75th)	Premovement % (25th-75th)	Movement % (25th-75th)	Return % (25th-75th)
Monkeys A & B	53.7 (26.5–71.8)	72.4 (39.6–76.7)	75.7(69.7–106.6)	70.4(40.4–73.5)
Monkey A	40.4 (20.2–73.2)	63.1(32.6–71.6)	73.6(71.5–106.6)	74.9(37.3–73.3)
Monkey B	59.4(41.2–71.7)	83.4(57.2–100.4)	93.4(67.3–113.7)	69.1(53.3–79.5)

Percentage ratios and ranges (25th-75th percentile) to values in cDBS were expressed.

## Discussion

The results confirmed the feasibility of a brain-machine interface in controlling the stimulus intervals and amplitudes of STN-DBS driven by the fast M1-γ2 oscillatory activity (Fig. 1E) in parkinsonian monkeys during a motor task. The M1-γ2 activity was robustly increased during the planning and execution of movement and successfully modulated the stimulus intervals and amplitudes of STN-DBS during task execution. The stimulus interval was minimized, the stimulus amplitude was maximized, and the dispersions of stimulus intervals and amplitudes were minimized during the task. Both aDBS and cDBS improved task performance over DBS-OFF, with aDBS delivering a lower electrical charge than cDBS.

There are several limitations of the present experimental paradigm that need to be acknowledged to adequately frame the discussion of our findings. One is the lack of feedback on side effects from DBS in an animal model. By comparison and in patients, cDBS is individually adjusted to optimize the benefits-to-side effects ratio because of the variations in anatomic structures and the extent of the electrical current spreading to adjacent structures^46^. The resulting side effects of the electrical current spread include sensorimotor, cognitive, and psychiatric effects^47–49^ which are generally subtle and verbally reported by the patients; such subtle events may be missed in studies with monkeys. In the current primate study, we carefully minimized the possibility for the occurrence of such side effects by using stereotactic guidance, electrophysiological mapping, and sensorimotor testing in each session to confirm the location of DBS lead contacts in the dorsolateral motor region of the STN. Also, our preliminary observations showed that cDBS at 150 Hz and over 2 mA occasionally induced side effects; therefore, we limited the frequency and amplitude for cDBS to 100 Hz and 1 mA, and those for aDBS to 50–150 Hz and 0.5–1.5 mA. A second limitation is the pulse width of 60 µs in both aDBS and cDBS. This duration of pulse width is commonly used in the clinical application of DBS; importantly, this short pulse width caused minor artifacts in M1-LFPs. This is likely due to the combination of optimizing the recording electrodes for common mode rejection, the short distance between paired electrodes, and the filters and sampling rate for LFPs recording. Longer pulse widths (> 200 µs) will likely require additional processing for the rejection of stimulus artifacts, which might cause reverberating effects in the close-loop system. For the reasons cited above, defining the optimal settings for cDBS (or aDBS) may be an elusive task in primate models. Therefore, we tested the two monkeys with the same stimulus parameters for the sake of the comparison between conditions and performed statistical analyses. To be sure, the present study may not be considered as a comparison of optimal clinical benefits between M1-γ2 aDBS and cDBS. Rather, this study supports the feasibility and compares the charge delivery between M1-γ2 aDBS and cDBS. Our data justifies further preliminary clinical studies during DBS surgical procedures in order to more formally compare between M1-γ2 aDBS and cDBS.

In fact, M1-γ2 activity successfully modulated the stimulus parameters of STN-DBS and improved PD motor function over DBS-OFF, with improved reaction and movement times, consistent with clinical benefits against bradykinesia and rigidity^50^. Elucidating the physiological mechanisms underlying the benefits from M1-γ2 aDBS is beyond the scope of this study. Nevertheless, our clinical observations may help to narrow the scope of future investigations aimed towards elucidating its mechanisms. One can start by looking at the kinetic profile of DBS therapy. Clinical benefits from DBS are known to show hysteresis^51^. When DBS is turned on or off in PD patients, changes in tremor, rigidity and bradykinesia are delayed by several seconds or minutes^52,53^ despite immediate changes in basal ganglia circuitry dynamics^51^. To be sure, we cannot exclude that these lag times may be decreased when DBS patterns are modulated rather than switched between ON and OFF states. Therefore, clinical benefits from aDBS may result from ‘near immediate’ effects from the stimulation when applied during movement. Alternatively, but not exclusively, clinical benefits from M1-γ2 aDBS during voluntary movement may result from delayed effects from stimulation delivered prior to the voluntary movement, i.e., during the Rest period and the preceding trials. In addition, and in contrast to cDBS, aDBS had dispersion in inter-stimulus intervals and amplitude. This finding on the train stimulation from aDBS is consistent with the nonstationary behavior of M1-γ2 activity during and between task periods. The dispersion in inter-stimulus intervals and amplitude during aDBS underlies irregular patterns in the stimulus pulse trains. Irregular train stimulations are known to provide clinical benefits comparable to those from cDBS but for different electrical charge^54,55^ in experimental and clinical applications^54,56–58^. In these regards, the present findings on M1-γ2 aDBS share similarities with the previous findings on irregular pulse train DBS. Therefore, dispersion features of stimulation pulses in aDBS may be another mechanism underlying the clinical benefits from M1-γ2 aDBS. Importantly, M1-γ2 aDBS showed lower electrical charge delivered during trials in comparison to cDBS. This effect was dependent on the task period, with the lowest relative charge delivered during the Rest period. An important clinical benefit from M1-γ2 aDBS may be to extend the battery lifetime by reducing electricity consumption when the patient is not engaged in motoric activity.

In line with studies focusing on other biomarkers^55^, the following two fundamental questions arise: (1) Is one biomarker better than the others to drive aDBS? (2) Is there a common neurophysiological mechanism underlying the clinical benefits emerging from these different adaptive systems? From our discussion and to answer these questions one will need to assess what are the features of the adaptive stimulation train that results in clinical benefits. A pivotal question is whether the correlation in time between the dynamic of the stimulation and the dynamic of the biomarker is necessary to the clinical benefits from aDBS. This should not be taken for granted since aDBS paradigms share similarity with other paradigms of stimulation such as the variable frequency paradigms^59^. The variable frequency paradigm is an alternative promising approach to aDBS with the presumption of DBS interacting with many intrinsic oscillators^60^ to reach broader circuitry effects and clinical benefits. Evidence for this last hypothesis is the dependency of PD symptoms on the frequency of STN-DBS^61^. For example, bradykinesia responds to stimulation over multiple different frequencies in a nonlinear manner^62^. The benefit of aDBS, comparatively to cDBS, may also result from the larger subset of frequencies multiplexed in the DBS pulse train. The point here is that there is uncertainty regarding the mechanisms underlying the clinical benefits from M1-γ2 aDBS and that future investigations will have to tackle the question of whether the correlation in time between stimulation’s dynamic and biomarker’s dynamic are necessary to explain the clinical benefits of aDBS.

This is certainly an important future direction rendered possible with our brain-machine interface. A possible approach would consist of using surrogate aDBS pulse trains for comparison to the original train stimulation. These surrogate aDBS pulse trains would share similar statistical characteristics to the original aDBS pulse trains, but they would be uncorrelated to the dynamic of the biomarker in time. Such surrogate DBS pulse trains can be generated by shuffling the order of the inter-pulse intervals of the original aDBS pulse train. Comparing the clinical benefits between by the original aDBS stimulation train and by its surrogate may provide valuable information on the mechanism by which aDBS alleviates motor symptoms in PD.

The present brain-machine interface is a tool for investigating aDBS driven by the cortical oscillatory activities in a given frequency band and its application is not limited to the γ2 band. By adjusting the filter setting, this system may be helpful in comparing the clinical benefits between different clinical markers used to drive aDBS. Our aDBS system required few setup parameters and it may be integrated easily into clinical settings such as in the operative room or possibly in clinic^63^. Although chronic intracortical recording is not commonly in use, technical difficulties have been overcome^64^, thus allowing such methodologies to move from clinical research to clinical application^65,66^. In this last case, and in PD patients, special attention should be paid to tremor episodes that are associated with increased γ band activity in the STN^19^ and possibly in M1 as well^67^. Since MPTP-treated macaque monkeys do not develop tremor, it remains to be established whether tremor-related activity may also benefit from M1-γ2 aDBS modulation. Also, possible interference may occur from dyskinesia, which may be associated with decreased γ1 activity^3,68^. Therefore, the divergence in cortical oscillations between voluntary movements and involuntary movements in the γ2 band should be evaluated as a factor that may predict clinical benefits in patients with PD and L-dopa-induced dyskinesia.

In summary, M1-γ2 aDBS is a safe strategy to positively modulate the stimulation train when therapeutic needs are presumed to be increased for facilitating voluntary movement. Our brain-machine interface allowed the delivery of higher pulse amplitude and higher pulse rate during the periods of preparation and execution of movement in comparison to the period of rest. M1-γ2 aDBS and cDBS had equivalent clinical benefits and M1-γ2 aDBS delivered only 2/3 of the charge of cDBS. Our study reports the feasibility and clinical benefits of M1-γ2 aDBS modulation in two parkinsonian monkeys. While our data are supportive for M1-γ2 aDBS modulation to require lower charge delivered than cDBS, the energy efficiency of M1-γ2 aDBS remains to be established in regard to the potential energy costs associated with running sensing and signal-to-stimulation processing in PD patients.

## Abbreviations

aDBS: Adaptive deep brain stimulation
cDBS: Constant deep brain stimulation
DBS: Deep brain stimulation
GPi: Internal pallidus
L-dopa: L-3,4-dihydroxyphenylalanine
LED: Light-emitting diode
LFP: Local field potential
M1: Primary motor cortex
MPTP: 1-Methyl-4-phenyl-1,2,3,6-tetrahydropyridine
PD: Parkinson disease
STN: Subthalamic nucleus
TTL: Transistor-transistor logic
